# Analyzing the Effects of Hyperspectral ZhuHai-1 Band Combinations on LAI Estimation Based on the PROSAIL Model

**DOI:** 10.3390/s21051869

**Published:** 2021-03-07

**Authors:** Yangyang Zhang, Jian Yang, Lin Du

**Affiliations:** 1 College of Resources and Environmental Sciences, Gansu Agricultural University, Lanzhou 730070, China; zhangyangyang@cug.edu.cn; 2 School of Geography and Information Engineering, China University of Geosciences, Wuhan 430074, China; dulin@cug.edu.cn; 3 Artificial Intelligence School, Wuchang University of Technology, Wuhan 430223, China

**Keywords:** leaf area index (LAI), ZhuHai-1 (ZH-1), band characteristic, Gaussian process regression (GPR), PROSAIL

## Abstract

Leaf area index (LAI) is a key biophysical variable to characterize vegetation canopy. Accurate and quantitative LAI estimation is significant for monitoring vegetation growth status. ZhuHai-1 (ZH-1), which is a commercial remote sensing micro-nano satellite, provides a possibility for quantitative detection of vegetation with high spatial and spectral resolution. However, the band characteristics of ZH-1 are closely related to the accuracy of vegetation monitoring. In this study, a simulation dataset containing 32 bands of ZH-1 was generated by using the PROSAIL model, which was used to analyze the performance of 32 bands for LAI estimation by using the hybrid inversion method. Meanwhile, the effect of different band combinations on LAI estimation was discussed based on sensitivity analysis and the correlation between bands. Then, the optimal band combination from ZH-1 hyperspectral satellite data for LAI estimation was obtained. LAI estimation was performed based on the selected optimal band combination of ZH-1 satellite images in Xiantao city, Hubei province, and compared with the Sentinel-2 normalized difference vegetation index (NDVI) values and LAI product. The results demonstrated that the obtained LAI map based on the optimal band combination of ZH-1 was generally consistent with the overall distribution of Sentinel-2 NDVI and the LAI product, but had a moderate correlation with Sentinel-2 LAI (R = 0.60), which may not favorably indicate the validity of indirect validation. However, the method of this study on the analysis of hyperspectral data bands has application potential to provide a reference for selecting appropriate bands of hyperspectral satellite data to estimate LAI and improve the application of hyperspectral data such as ZH-1 in vegetation monitoring.

## 1. Introduction

Leaf area index (LAI) as a common parameter of vegetation is usually expressed as one half of the total green leaf area per unit of ground surface area [1], which can effectively reflect the processes of photosynthesis, respiration, transpiration and so on [2,3]. It is also an essential data source for precision agriculture such as crop growth monitoring, crop yield estimation, and fertilizer management. Therefore, it is of great significance to accurately estimate crop LAI and its dynamic changes for agricultural study and application [4]. Traditional LAI measurement methods are time-consuming and laborious, and can only obtain LAI information on scattered sample points, which is difficult to carry out on large scales and long time series, and its timeliness is difficult to guarantee [5].

Compared to traditional methods, remote sensing technology is a valid method to estimate LAI due to its fast, non-destructive and large-scale advantages. At present, remote sensing data has been successfully applied in many studies for LAI estimation [6,7,8,9,10,11]. Currently, empirical and physical methods are the two most common types of LAI estimation based on remote sensing data [12,13,14]. The empirical methods usually use LAI measurements and spectral data to construct linear or nonlinear relationships [15,16], which have the advantages of simplicity, fast and few input parameters, but usually lack generality and the physical mechanism [17,18]. The physical methods are implemented through the radiation transfer model (RTM), which are generalizable to different vegetation types and background environments, but the implementation and application process is complex [19,20]. Among various RTMs, the PROSAIL model was widely used for its simplicity and accuracy. Moreover, its reliability has been tested with different types of datasets [21]. When run in forward mode, the PROSAIL model can be used for the generation of simulation datasets [22]. Currently, iterative optimization, look-up table (LUT) and hybrid inversion are the three strategies for vegetation parameter estimation using physical methods [23,24]. The first inversion method is classical but its calculation is heavy, and its convergence is poor [23]. While the LUT method is a valid alternative, different optimization studies of LUT inversion were performed for different biophysical parameters and sensors [25]; the optimal cost function for the ZhuHai-1 (ZH-1) sensor is still unknown. The limitations of these methods can be overcome by proposing a hybrid inversion method that generates a simulation database using the RTM and then estimates vegetation parameters using machine learning algorithms [24]. The hybrid inversion method combines the simplicity of the empirical approach with the generalizability of the physical model to accurately and quickly estimate vegetation physicochemical parameters, such as LAI, chlorophyll content, etc. [26,27]. Hybrid inversion methods can be combined with the RTM using different machine learning algorithms [28,29,30,31,32,33], such as artificial neural networks (ANN), support vector regression (SVR), random forest (RF), Gaussian process regression (GPR), and so on. RF was applied to the estimation of vegetation biomass [34]. ANN and RF had been used for estimation of crop LAI using hyperspectral vegetation indices [22]. Tuia et al. presented a multi-output version of SVR, along with estimations of LAI and chlorophyll content [35]. Of all the machine learning regression algorithms, the best performance was probably Gaussian process regression (GPR) [36]. GPR has only recently been applied to remote sensing spectral data, for example, for chlorophyll content mapping in HyMap [37], and for LAI mapping in CHRIS [28]. Rivera Caicedo et al. [38] confirmed that GPR outperformed most machine learning algorithms in estimating leaf chlorophyll content and LAI for different spectral datasets. Meanwhile, Ashourloo et al. [39] demonstrated that GPR was the most accurate in detecting leaf rust; it was found more accurate than partial least squares regression (PLSR) and SVR. Therefore, GPR was used to analyze the performance of the LAI estimation by using ZH-1 hyperspectral satellite data in this study.

Currently, remote sensing satellites have been developed rapidly, and sensor accuracy has been greatly improved. However, to enhance land surface monitoring, spectral, temporal and spatial resolution need to be improved. The ZH-1 satellite constellation is a commercial remote sensing micro-nano satellite constellation that was launched and operated by a private listed company in China. The entire constellation consists of 34 satellites, including video satellites, hyperspectral satellites, radar satellites, high-resolution optical satellites and infrared satellites. Among them, there are 4 Orbita hyperspectral satellites (OHS): spatial resolution of 10 m, image range of 150 × 2500 km, 32 bands, spectral resolution of 2.5 nm and spectral range of 40–1000 nm. Moreover, the four hyperspectral satellites of ZH-1 are important members of the global family of 25 hyperspectral satellites and are the only commercial hyperspectral satellites that have been launched and networked in China. The characteristic hyperspectral data are capable of precise and quantitative analysis of vegetation, water, ocean and other features, fully reflecting the advantages of quantitative satellite remote sensing. Therefore, it is necessary to explore the characteristics of ZH-1 satellite bands for retrieving and monitoring the important biophysical parameter of LAI. In this study, the performance of 32 bands and different band combinations of ZH-1 hyperspectral data for LAI estimation by using the hybrid inversion method is discussed. However, hyperspectral data typically include highly correlated and noisy spectral bands, and the potentially redundant spectral bands can affect the accuracy of predictions and the interpretability of regression (retrieval) models [30]. For hyperspectral data, the selection of an appropriate band combination has important implications for both model construction and vegetation parameters estimation [22,40]. To exploit the potential of hyperspectral data, the selection of sensitive bands to form band combinations was recommended for improving the accuracy of vegetation variables estimation. In order to obtain an accurate LAI estimate, it is crucial to screen the appropriate band combination. The ideal band combination should be sensitive to inversion parameters and insensitive to interference factors [22]. Therefore, evaluating the performance of each band of ZH-1 hyperspectral data for LAI estimation and selecting bands that can accurately invert LAI are crucial for the application of ZH-1 data in quantification analysis of vegetation. Although there are many studies of spectral band selection for hyperspectral data, most of them are related to classification problems [41,42], and few are concerned with regression (retrieval) problems, especially vegetation parameter (for example, LAI) estimation. Hence, sensitivity analysis was used to evaluate the relationship between spectral bands of ZH-1 hyperspectral data and LAI, and obtained the optimal band combination for accurate estimation of LAI in the regression model in this study. Meanwhile, it provided a reference for the spectral band selection for other hyperspectral data in vegetation parameter estimation.

In this study, the PROSAIL model was used to generate simulated datasets based on the spectral response function of 32 channels of ZH-1 hyperspectral satellite. The effects of different band combinations on LAI estimation were discussed based on sensitivity analysis and the correlation between bands. Then, the optimal combination of spectral bands for LAI estimation based on ZH-1 hyperspectral satellite was analyzed by using GPR. LAI of the study area was estimated based on the optimal band combination, and Sentinel-2 normalized difference vegetation index (NDVI) and LAI products were used for preliminary verification. The band characteristics and optimal bands selection for LAI estimation using ZH-1 hyperspectral satellite were evaluated, and a reference was provided for the application of ZH-1 for vegetation monitoring.

## 2. Materials and Methods

### 2.1. Study Area

**Figure 1:**
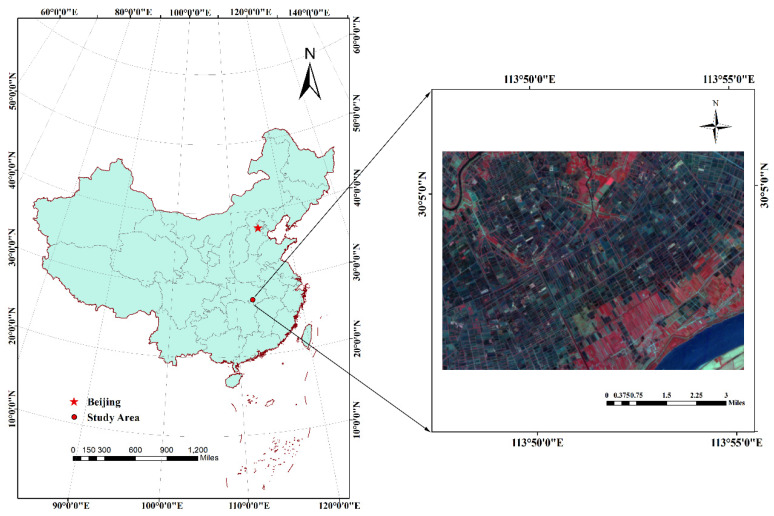
Location of the study area ZhuHai-1 (ZH-1) image shown on the right side of this figure with false color composite: R = near infrared, G = red, B = green).

The study area is the Xiantao city in south-central Hubei province, China, located in the Jianghan plain (112°55′–113°49′ E, 30°04′–30°32′ N), which is 78 km long from east to west, 35 km wide from north to south, and has a total area of 2538 km^2^. Xiantao city belongs to the subtropical monsoon climate zone, with a mild climate, abundant rainfall, sufficient sunshine, four distinct seasons and a long frost-free period [43]. Average annual rainfall is 1215.0 mm, the annual average sunshine hours are 2002.6 h, the sunshine rate is about 46%, the annual average temperature is 16.3 °C, and the frost-free period is generally 256 days, which is conducive to the growth of crops [44]. The main land use types in the study area are cropland, forest and water, which account for about 45%, 15%, and 5%, respectively, while villages are also included and the main crop types are rice and oilseeds. The location of the study area is shown in Figure 1.

### 2.2. Remote Sensing Data

The ZH-1 satellite constellation is a commercial remote sensing micro-nano satellite constellation that was launched and operated by a private listed company in China. The ZH-1 satellite constellation consists of 34 satellites, including video satellites, hyperspectral satellites, radar satellites, high-resolution optical satellites and infrared satellites. The OHS was successfully launched on 26 April 2018, which was capable of accurate quantitative analysis of vegetation, water, ocean and other ground objects. The ZH-1 data was downloaded from the official Orbita Hyperspectral Satellite website (www.obtdata.com; Zhuhai Orbita Aerospace Science & Technology Inc., Zhuahai, GD, China), which was a commercial satellite resource.

The OHS2A_CMOS3 data of ZH-1 hyperspectral satellite, with a spatial resolution of 10 m and a total of 32 spectral bands, were obtained on 16 April 2019. The actual size of the study area was 50.56 × 50.56 km. To convert the digital number (DN) of the raw image to land surface reflectance, the ENVI 5.3 software was used to preprocess the ZH-1 image in this study, including radiometric calibration, atmospheric correction and geometric correction. Radiometric calibration was carried out by the ZH-1 absolute calibration coefficient, which converted the DN value of the raw image into absolute radiation value. Atmospheric correction was carried out by the FLASSH atmospheric correction module of ENVI 5.3 to obtain the land surface reflectance. The imaging location was Xiantao city, Hubei province, China and the imaging area features towns, rivers, farmland and so on. The band settings of ZH-1 hyperspectral data are shown in Table 1.

### 2.3. Workflow

**Figure 2:**
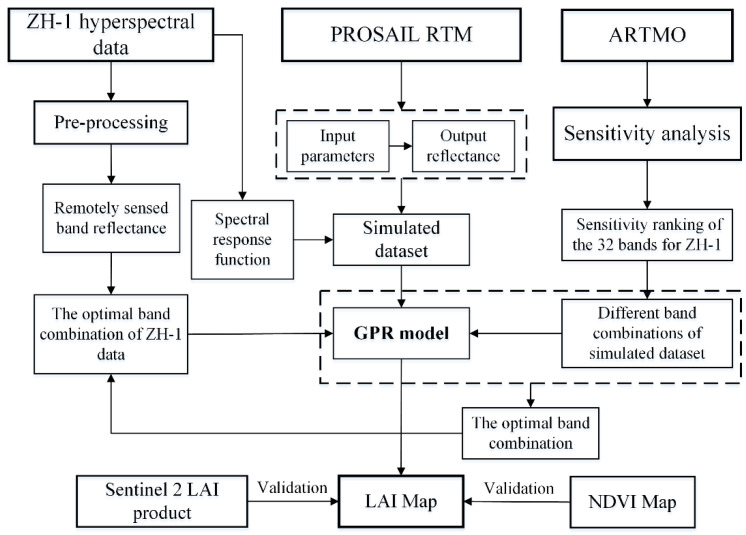
The workflow of this study.

Figure 2 shows the workflow chart of this study. In this study, the band characteristics and application potential of ZH-1 for LAI estimation by using the GPR algorithm combined with PROSAIL was analyzed. Firstly, a simulated dataset with ZH-1 band characteristics was generated based on the PROSAIL model, which was used as a training dataset for the GPR model. Then, sensitivity analysis was calculated for the spectral range of ZH-1 satellite data (400–1000 nm). The band was added sequentially to form different band combinations to estimate LAI based on the band sensitivity ranking, and the optimal band combination for LAI estimation was selected. Finally, an LAI map of the study area was obtained by the optimal band combination of ZH-1 hyperspectral data. The main vegetation type in the study area was crop, this study verified the application of ZH-1 data for crop LAI estimation, and the vegetation type will be expanded for the further study.

### 2.4. Gaussian Process Regression

GPR is essentially a non-parametric model of using Bayesian inference. GPR uses the Gaussian process as a priori; it assumes that the learning sample is a sample of the Gaussian process and its estimates are closely related to the kernel function. The practical meaning of the kernel function in GPR is the covariance function, which describes the correlation between learning samples and is not a means of simplifying computation through the kernel approach, but is part of the model assumptions. The essence of Gaussian regression is actually a mapping of the independent variables from a low-dimensional space to a high-dimensional space, and the probability distribution can be obtained with the appropriate kernel function. Gaussian regression first calculates the joint probability distribution between samples in the dataset, and then calculates the posterior probability distribution of the predicted values based on the prior probability distribution of the predicted values. In this study, a squared exponential kernel function was used

(1) $$k(x_{i},x_{j})=\exp(-\frac{∥x_{i}-x_{j}{∥}^{2}}{2\sigma^{2}}),$$

which extracted well to sample similarity in most problems and only required adjustment of a hyperparameter *σ*. The usual approach to solving the hyperparameter was to use the edge likelihood function to derive the model parameters, and finally to use the conjugate gradient method to obtain the edge likelihood maximum to solve the hyperparameter. More details of GPR can be referred to in [28,45,46]. The GPR model was implemented by using MATLAB R2015B (Mathworks Inc., Natick, MA, USA).

In this study, the GPR model was used to estimate LAI using a simulated dataset of canopy spectral and corresponding vegetation parameters generated by the PROSAIL model as the training dataset. LAI values estimated by the GPR method may have outliers; when the LAI value estimated by the GPR method was less than 0 or greater than 10, it was considered as an outlier. For an outlier pixel, the mean of its eight neighbor pixel values was used as the new pixel value for that outlier pixel. GPR was an LAI estimation for each individual pixel and did not consider the correlation between pixels. There was pixel noise in the ZH-1 image, and there may be some discrete point noise in the obtained ZH-1 LAI map. Therefore, mean filtering in a 3 × 3 window size was used for smooth denoising of the ZH-1 LAI map.

### 2.5. PROSAIL Model

PROSAIL RTM is a coupled model of PROSPECT leaf optical properties model [24] and SAIL canopy reflectance model [47]. Currently, various versions of the PROSAIL model have been developed, PROSAIL5B, a combination of PROSPECT5 and 4SAIL, was used to simulate vegetation canopy reflectance spectra under different conditions [48,49]. Using the randomly generated input parameters by running the PROSAIL model in forward mode, it is possible to generate vegetation canopy reflectance simulation datasets with spectral intervals of 1 nm in the spectral range of 400–2500 nm, and the simulation datasets generated by the PROSAIL model can contain different vegetation types under different conditions. To simulated sensor noise, 2% random Gaussian noise was added to the canopy reflectance of the simulated dataset. This study mainly analyzed LAI estimation of ZH-1 hyperspectral data. Therefore, according to the spectral response function and the range of each band of ZH-1 hyperspectral data, the simulated datasets were generated by the PROSAIL model. The key input parameters of PROSAIL are summarized by many literatures in Table 2 [22,50,51,52,53,54], where N: structure index, Cab: chlorophyll, Car: carotenoid, Cw: equivalent water thickness, Cm: dry matter per area, LAI: leaf area index, ALIA: average leaf inclination angle, hspot: hot-spot parameter, and psoil: soil brightness factor. The hspot parameter was introduced to correct for the hotspot effect problem of the PROSAIL model, which was generally expressed using the ratio of leaf size to canopy height [21]; the psoil parameter characterized the degree of soil dryness (psoil = 1) and wetness (psoil = 0), and N actually described the internal structure of the leaf.

Hence, the input parameters of PROSAIL and the spectral response function of the ZH-1 satellite were used to generate the simulated dataset. The reflectance information of the simulated dataset was used as input to the GPR model, and the corresponding LAI parameter was used as output to train the GPR model. A squared exponential kernel function was used as the kernel function of the GPR model and the other parameters were default values. Then, LAI estimation was made by the trained GPR model using correlation bands from ZH-1 satellite data through sensitivity analysis. The simulated datasets, which had size of 5000, were used for training and verification of the GPR, among which 2500 datasets were used as the training dataset of GPR for LAI estimation, and the rest were used as verification datasets for accuracy verification of the trained model.

### 2.6. Sensitivity Analysis and ARTMO

Sensitivity analysis (SA) is the calculation of the contribution of a given input variable to the variance of an output variable. The influence of input parameters, especially LAI, on canopy reflectance can be obtained by SA of PROSAIL, as well as the list of bands sensitive to LAI changes within the band range of 400–2500 nm. In this study, the spectral range of the ZH-1 satellite data (400–1000 nm) was analyzed and Automated Radiative Transfer Models Operator (ARTMO) was used to complete sensitivity analysis [55]. ARTMO contains a set of leaf and canopy RTMs, including PROSAIL and several retrieval toolboxes. The ARTMO toolbox runs in MATLAB and can be downloaded for free from http://ipl.uv.es/artmo/.

### 2.7. Validation and Statistical Evaluation

Due to the lack of field measurements in the study area, it is not possible to directly quantitatively evaluate the accuracy of the LAI estimation. NDVI is the most commonly used vegetation index to reflect vegetation distribution [56]. Some studies described the correlation between NDVI and LAI, and estimated LAI by NDVI [5,57,58,59,60,61]. Therefore, this study proposed to validate the accuracy of the distribution of LAI estimates using NDVI. NDVI is the ratio of the difference between and the sum of the near-infrared (NIR) and red bands and is the most commonly used index for LAI estimation [62]. The ZH-1 LAI map and Sentinel-2 NDVI map were used to evaluate the accuracy of LAI estimation indirectly and quantitatively. Meanwhile, the Sentinel-2 LAI product was used for comparison to the ZH-1 LAI estimation. Using the Sentinel-2 Land bio-physical processor (SL2P) within the SNAP Toolbox, the Sentinel-2 LAI/NDVI map with a spatial resolution of 10 m can be obtained, which matched the spatial resolution of the ZH-1 LAI map. The Sentinel-2 satellite image on 8 April, 2019 was acquired from ESA Copernicus Open Access Hub (https://scihub.copernicus.eu/dhus/). More details on SL2P can be found in [63]. In addition, MOD15A2 of MODIS-LAI product data were also used to verify the accuracy of LAI estimation directly. The acquisition time of the MOD15A2 product was from 15 April, 2019 to 22 April, 2019, which was consistent with the acquisition time of the remote sensing data. The spatial resolution of MOD15A2 was 500 m, while the spatial resolution of ZH-1 LAI map was 10 m, hence, the LAI map obtained from the resampled ZH-1 data was used for accurate comparison with MOD15A2. Since the MOD15A2 resolution was too low, a larger area was selected for comparison. Similarly, the corresponding pixels were still randomly selected from the ZH-1 LAI map and the MODIS-LAI map, and the accuracy of LAI estimation was quantitatively evaluated by analyzing the correlation between these randomly selected pixels. The LAI values of each pixel were randomly selected in different distribution regions throughout the study area.

To evaluate the performance of different bands for the LAI estimation based on GPR, the determination coefficient (R^2^) and root mean square error (RMSE) were used as model accuracy indexes. The closer R^2^ is to 1, the higher the fitting accuracy of the model is, and the smaller the RMSE is, the smaller the difference between the predicted value and the measured value, the better the predictive power of the model is. The predicted and measured values of the verification set were analyzed, and the ability of different bands and band combinations to use GPR model for LAI estimation was evaluated. Finally, the band combined with the highest accuracy was selected as the optimal input of the GPR model to estimate the LAI of the study area.

## 3. Results

### 3.1. Sensitivity Analysis of PROSAIL Model

**Figure 3:**
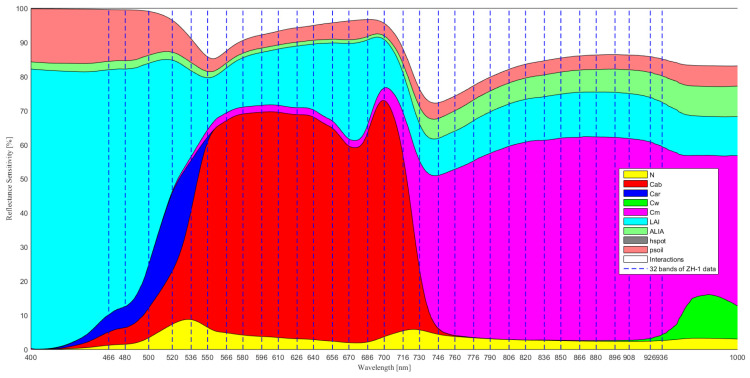
Global sensitivity analysis (GSA) of the PROSAIL model for canopy reflectance (The dashed lines represent the 32 bands of ZH-1 data. N: structure index, Cab: chlorophyll, Car: carotenoid, Cw: equivalent water thickness, Cm: dry matter per area, LAI: leaf area index, ALIA: average leaf inclination angle, hspot: hot-spot parameter, psoil: soil brightness factor. Table 2 shows the application units and input parameter ranges for GSA).

Different parameters of the PROSAIL model contributed differently to canopy reflectance in different spectral regions. In Figure 3, the contributions of LAI and other parameters were shown in different colors according to the SA results and the dashed lines represented the 32 bands of ZH-1 data. From Figure 3, it can be seen that the LAI parameter had a distinctly sensitive spectral domain, and its influence was significantly stronger than other parameters in some spectral ranges. Meanwhile, the sensitivity list of the 32 bands based on the ZH-1 satellite data to the LAI parameter was obtained.

According to the SA results of each input parameter of PROSAIL, combined with the location of 32 bands in the ZH-1 hyperspectral data, the sensitivity list of the 32 bands based on the ZH-1 satellite data to the LAI parameter was obtained. 

### 3.2. Consistency Comparison between Single-Band LAI Estimation and Sensitivity Ranking

**Figure 4:**
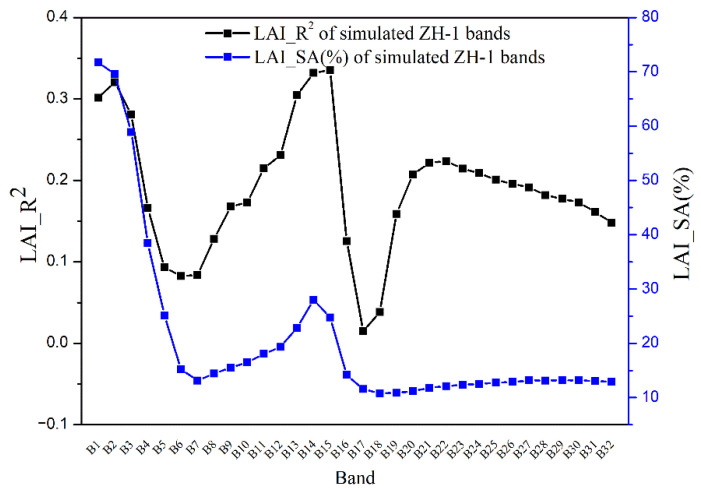
Single-band LAI estimation and sensitivity comparison.

Before selecting bands to invert LAI based on sensitivity, it is necessary to determine whether the sensitivity of 32 bands to LAI is consistent with the accuracy of LAI estimation. Figure 4 shows the results of LAI estimation using GPR for the simulated dataset.

It can be seen that different bands have different effects on the LAI estimation, and LAI estimation varied greatly among different bands. For example, the difference between R^2^ and RMSE of the B2 and B6 bands can reach 0.24 and 0.26, respectively, indicating that the effective information of LAI estimation provided by different bands differed greatly. The B17 band had the minimum R^2^ and the maximum RMSE, the B15 band had the maximum R^2^ and the minimum RMSE. Moreover, the performance and sensitivity of each band for LAI estimation is also shown in Figure 4. The variation tendency of LAI estimation accuracy based on the single band in the 32 bands was consistent with the variation tendency of band sensitivity (Figure 4). Therefore, it was feasible to determine the performance of bands for LAI estimation based on the sensitivity analysis of 32 bands.

### 3.3. Analysis of LAI Estimation with Different Band Combinations

According to the ranking of sensitivity shown in Figure 4, the bands which had a greater impact on LAI change were selected according to the sequence of sensitivity for LAI estimation. From the B1 band with the highest sensitivity, highly sensitive bands were added successively to form a band combination to estimate LAI. Lastly, LAI estimation results of 31 band combinations were added successively from the combination of B1B2 with the highest sensitivity to B19. When the number of band combinations is too high, it may cause reading inconvenience. Hence, the values of the number of band combinations were used to represent the specific band combinations as shown in Table 3. For example, C7 in Table 3 represented the B1B2B3B4B14B5B15 band combination.

**Figure 5:**
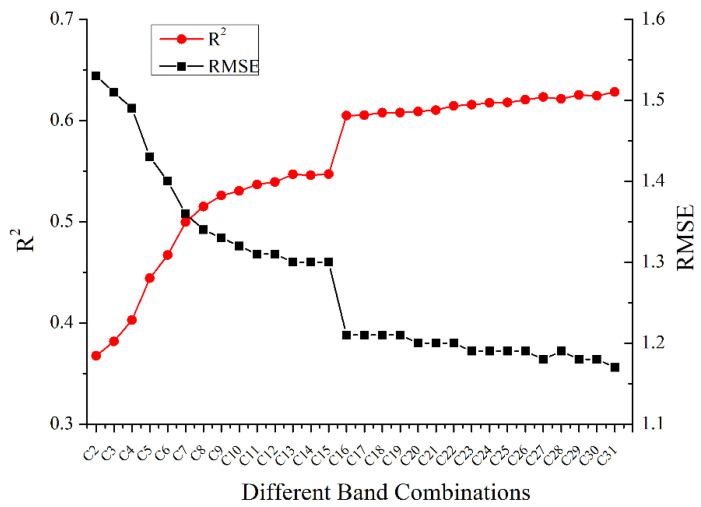
LAI estimation of the different number of combined bands.

As can be seen from Figure 5, the number of added bands was not proportional to the inversion accuracy of LAI, which may be because the redundant information among bands increased with the number of bands, and the increased noise among bands would limit the improvement of LAI estimation. At the same time, along with the increase in inversion bands, LAI inversion results of different band combinations showed obvious differences. Hence, when multiple bands were used to estimate LAI, the accuracy of LAI estimation based on different band combinations should be compared to select the optimal inversion method.

The C5 band combination and the C16 band combination had a significantly higher improvement in LAI accuracy than the other band combinations. As can be seen from Figure 5, the accuracy did not improve steadily with the increase of the number of bands, and different added bands had different effects on the improvement of LAI accuracy. This indicated that in the process of adding bands from C2 to C31, the added bands had different effects on LAI estimation, and some additional bands cannot provide effective information for LAI estimation and may reduce the performance of the previous band combination. After adding B29, the inversion accuracy of C16 was greatly improved, which may demonstrate that B29 provided very critical and effective spectral information for LAI estimation. Hence, based on the LAI estimations of band combinations and the improvement in the accuracy of LAI estimation of each additional band, the band combination B1B2B4B14B5B15B13B29B19 was finally determined as the optimal band combination for LAI estimation.

### 3.4. Analysis of LAI Estimation of the Optimal Band Combination

LAI estimation was performed by using the selected optimal band combination and the results were compared with those of the full band combination, as shown in Table 4. The coefficient of variation was used to represent the relative uncertainty of the GPR model [64]. A larger CV indicated a greater degree of dispersion, higher model uncertainty, and greater prediction risk, and vice versa.

Table 4 shows that the differences between the inversion accuracy and CV value of the full band combination and the optimal band combination were small, indicating that the optimal band combination of ZH-1 hyperspectral satellite data had great potential in LAI estimation. The results also showed that more bands were not better for LAI estimation, and the number of bands was not always proportional to the accuracy. Moreover, full band combination may result in large amounts of information redundancy between the adjacent bands. Spectral information also included background noise which will greatly limit the improvement of LAI estimation accuracy using full band combination.

The first seven bands in the optimal band combination B1B2B4B14B5B15B13B29B19 were the bands with the highest sensitivity to LAI, and B14B15 belonged to the red-edged band, which was closely related to LAI. The correlation between the optimal band combination and LAI was calculated based on simulated dataset and is shown in Table 5. From Table 5, the correlation between B29/B19 and the other seven bands was low, and the other seven bands show a good negative correlation with LAI, while B29 and B19 show a positive correlation with LAI. Thus, B29 and B19 might contain some useful information which was conductive to LAI estimation.

### 3.5. Validation of the LAI Map of ZH-1 Data

**Figure 6:**
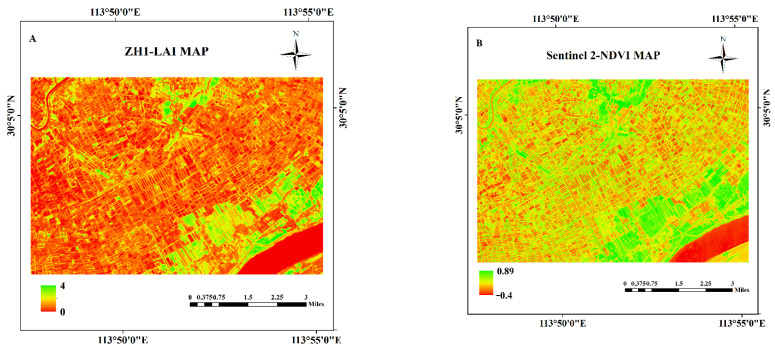

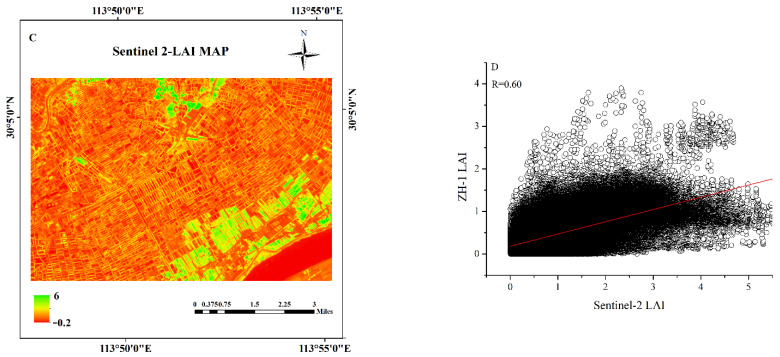
(**A**): LAI map of the study area using the optimal band combination of ZH-1 hyperspectral satellite; (**B**): NDVI map of the study area with Sentinel-2 image; (**C**): LAI map of the study area using Sentinel-2 hyperspectral satellite; and (**D**): correlation between ZH-1 LAI and Sentinel-2 LAI.

The optimal band combination of ZH-1 hyperspectral data for LAI estimation was used to invert LAI for the study area using the GPR model, and the LAI map of the study area is shown in Figure 6.

The study area was located in the Jianghan plain with a suitable climate and sufficient water, which was conducive to the growth of crops. It can be seen from Figure 6A that the overall distribution of LAI in the southern part of the study area was high, which was consistent with the vegetation growth of the study area. The LAI value in the central part of the study area was significantly lower than that in other areas.

Meanwhile, NDVI calculated from Sentinel-2 data of the study area is shown in Figure 6B. As can be seen from Figure 6A,B, the distribution of LAI is broadly consistent with that of NDVI. The central LAI and NDVI distributions are both relatively low in the study area, and the southeast and partly northern LAI and NDVI distributions are higher than the other areas, while relatively low LAI and NDVI are shown in the eastern of the study area. According to the NDVI map, the LAI map in the study area performed similar regional distribution trends, suggesting that the LAI estimation obtained by the optimal band combination of ZH-1 hyperspectral data was feasible.

The Sentinel-2 LAI map of the study area was obtained (Figure 6C) by using the Sentinel-2 Land bio-physical processor (SL2P) within the SNAP Toolbox. The overall distribution of LAI as shown in Figure 6A,C was generally consistent, with both showing that some regions in the southeast and north have significantly higher LAI values than other regions. However, the Sentienl-2 LAI values ranged from −0.2 to 6 and ZH-1 LAI values ranged from 0 to 4, and the two LAI distributions were not quite consistent. Negative values presented in the Sentinel-2 LAI can be considered outliers. Furthermore, some studies indicated that SNAP-derived LAI may significantly overestimate LAI values for different crops [65,66]; the comparison of the ZH-1 LAI map with the Sentinel-2 LAI map also confirmed this conclusion.

Corresponding pixel points from the ZH-1 LAI map and the Sentinel-2 LAI map were used to evaluate the accuracy of LAI estimation indirectly and quantitatively. The LAI estimation generated by the optimal band combination of ZH-1 data, compared to the LAI from Sentinel-2 data, had a correlation of R = 0.60 (Figure 6D). ZH-1 LAI values were lower than Sentinel-2 LAI values, which may be partly due to the overestimation of Sentinel-2 LAI, and partly due to the lack of a priori knowledge of the study area for the input variables in the inversion algorithm. The relationship between ZH-1 LAI and Sentinel-2 LAI indicated that the optimal band combination of ZH-1 produced reasonable LAI estimations, but there were also significant underestimates and saturation plateaus.

**Figure 7:**
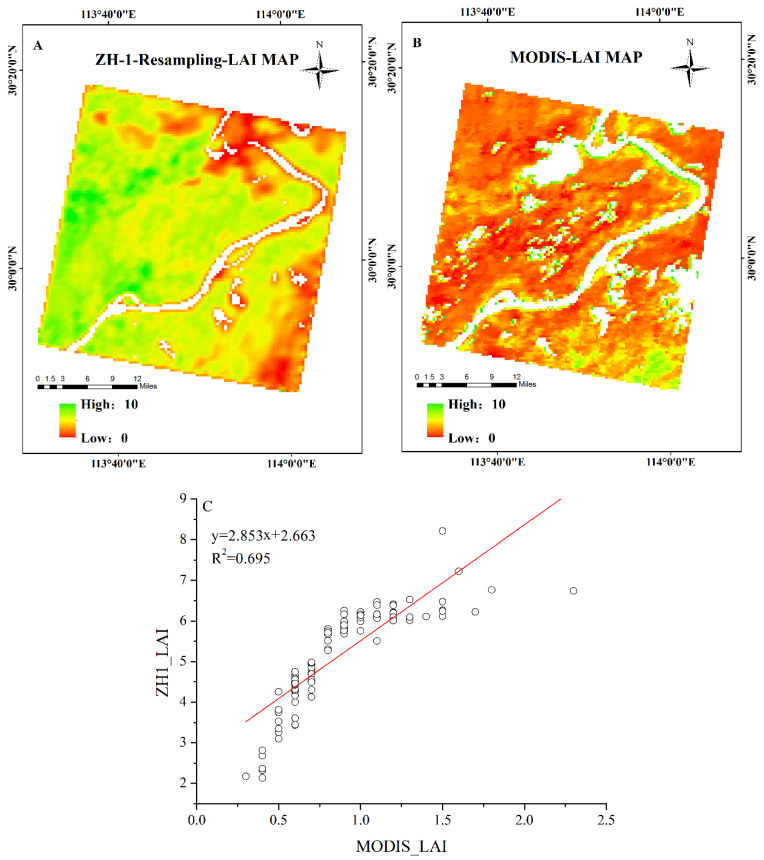
(**A**): LAI map of the study area using the optimal band combination of resampled ZH-1 hyperspectral satellite; (**B**): MODIS LAI map of the study area; and (**C**): correlation between the ZH-1 LAI estimation and the MODIS LAI for randomly selected pixels from the ZH-1 LAI map and the MODIS-LAI product, respectively.

The optimal band combination of resampled ZH-1 hyperspectral data was used for LAI estimation, and the LAI map of the study area is shown in Figure 7A. Meanwhile, Figure 7B shows the MODIS LAI map of the study area to perform a direct comparative validation of the ZH-1 LAI map.

As can be seen from Figure 7A,B, ZH-1 LAI values were significantly higher than MODIS LAI values throughout the study area, which may be due to the tendency of the MODIS LAI product to underestimate LAI in wet regions [67]. This was due to the low spatial resolution of the MODIS data, making many crop areas of MODIS pixels become mixed pixels, including road, water and buildings and other feature types. These feature types especially in the NIR reflectance were much lower than the crop canopy reflectance, making the overall reflectance of the mixed pixels low, resulting in low MODIS LAI pixel values [68]. However, the northern region all exhibited a low distribution of LAI and the southern and central regions had relatively high LAI compared to the other regions. To quantitatively evaluate the accuracy of LAI estimation, correlation between 103 corresponding pixels randomly selected from the ZH-1 LAI map and the MODIS LAI product were analyzed (Figure 7C). LAI estimates generated from the optimal band combination of resampled ZH-1 data compared to the MODIS LAI product had a good correlation of R^2^ = 0.695 for the 103 randomly pixels. The apparent correlation suggested that LAI estimation of the optimal band combination of ZH-1 data was reasonable. Tthe slope of the linear relationship clearly indicated an underestimation of MODIS LAI product, where the ZH-1 LAI may also be overestimated. In Figure 7B and C, the MODIS LAI product exhibited a clear vertical distribution characteristic, which indicated that more continuous pixels in the MODIS LAI product presented the same LAI values. Considering the coarse spatial resolution of MODIS LAI product and the growth status of vegetation in the study area, it may not be practical to have the same LAI value over a wide area.

## 4. Discussion

In this study, the performance of 32 bands and different band combinations of ZH-1 hyperspectral data for LAI estimation by using the hybrid inversion method was discussed. For the hybrid inversion method, a combination of machine learning regression algorithms (MLRAs) and physical models was used to estimate vegetation parameters. The hybrid inversion method needs a small number of in situ measurements for accuracy validation, while model training can be performed with the simulated dataset generated by RTM. Due to the generalizability of RTM, once a suitable inversion model has been established, it can in principle be used for similar vegetation types acquired by different sensors [21]. Durbha et al. used an SVR model trained on simulated data from the PROSAIL model to retrieve the LAI from the MISR data [29]. And RF was also used for LAI and LCC estimation after training through the PROSAIL model generated dataset [29,69]. Verrelst et al. performed the inversion of biophysical variables for the same dataset using parametric, nonparametric, and physical model-based retrieval methods, respectively, and quantitatively evaluated the estimation accuracy and processing speed of the three types of methods [64]. The results showed that the processing speed and accuracy of LAI retrieval using GPR were better than other types of inversion methods. And, this study also showed that crop LAI can be effectively retrieve by GPR using hyperspectral data. GPR with a Bayesian framework has advantages over these MLRAs for vegetation parameters inversion, but all studies on GPR are still in experimental stage

For wavelength selection of hyperspectral data, to obtain higher inversion accuracy when estimating vegetation parameters from hyperspectral data, several studies have used different inversion methods to construct relationships between full-spectrum reflectance and the corresponding biophysical parameters [23,26]. However, the use of full-spectrum as the input parameter has limitations: the uncertainty of the effect of bands on vegetation parameter estimation and the redundancy of information between adjacent bands make the inversion model computationally complex and difficult to interpret. Meanwhile, traditional inversion of vegetation parameters using a single red-NIR band could not take full advantage of spectral information from hyperspectral data, and the single band performed poorly in vegetation parameter (for example, Figure 4) [30]. Sun et al. proposed characteristic wavelength selection algorithm for leaf chlorophyll content and leaf water content estimation using leaf spectral data, which was based on the results of SA and band-to-band correlation analysis for characteristic wavelength selection [70]. It indicated that wavelength selection using SA and correlation analysis was feasible for the estimation of vegetation parameters. The analysis of Figure 5 in this study suggested that simply combining bands that were sensitive to LAI may not get the highest inversion accuracy. A high degree of redundancy and correlation in the band combinations may reduce the accuracy of LAI estimation. When estimating LAI or other vegetation parameters from hyperspectral data using hybrid inversion methods, the redundancy and correlation of band combinations, namely interference resistance, must be analyzed.

The red-edge and NIR bands were found to be the most sensitive bands for LAI retrieval, the optimal band combination of ZH-1 hyperspectral data for LAI estimation also included these bands, which was consistent with previous studies [71,72,73]. In addition, the ZH-1 hyperspectral satellite was the only commercial hyperspectral satellite that has been launched and networked in China. Kganyago et al. compared the Sentinel-2 LAI product from the semi-arid agricultural landscape in Africa with global LAI products and showed that the R^2^ values of Sentinel-2 LAI product and measured LAI could reach about 0.6–0.7, but the errors of RMSE and BIAS were relatively large, while the R^2^ between theSentinel-2 LAI product and MODIS and Proba-V LAI products is about 0.55–0.8, and the RMSE error is small [65]. Hence, Sentinel-2 LAI product has some implications for characterizing LAI of agricultural crops. Due to the lack of ground measured data, ZH-1 LAI map was indirectly validated against the Sentinel-2 LAI product. This indirect validation partly demonstrates the feasibility of using the optimal band combination of ZH-1 data, but as seen in Figure 6D, ZH-1 LAI severely underestimates the Sentinel-2 LAI product and there is a saturation plateau around the LAI value of 2, which makes the indirect validation potentially uncertain. However, the proposed method is important in analyzing the sensitive bands of ZH-1 hyperspectral data in LAI inversion, obtaining the optimal band combination for LAI estimation, and the band selection of other hyperspectral data in vegetation applications. The absence of ground measured data validation is still a deficiency that needs to be filled at the future work.

## 5. Conclusions

The band characteristics of the satellite are closely related to the accuracy of LAI estimation. To determine the optimal spectral band combination for estimating vegetation properties from ZH-1 hyperspectral data, the band characteristics and application potential of ZH-1 for LAI estimation by using GPR algorithm combined with PROSAIL was analyzed in this study. The results of the study indicated that different band combinations had a great influence on LAI estimation and selecting an appropriate band combination can effectively improve the accuracy of LAI estimation based on ZH-1 data. Hence, the selection of an appropriate band combination in hyperspectral data was key to improving the accuracy of LAI estimation. Although the indirect validation was not successful based on the current data, this study had a high potential for application in band selection for LAI estimation using hyperspectral data. ZH-1 data can be applied to the determination of the extent of deforestation as forests are declining due to anthropogenic pressure, etc. However, due to the lack of field measurements, the accurate verification of LAI estimation obtained from ZH-1 needs to be improved in further study.

## Figures and Tables

**Table 1:** ZH-1 hyperspectral satellite data band settings.

Band	Spectrum Type	Wavelength/nm	Start/nm	End/nm	Bandwidth/nm
B01	Blue	466	464	468	4
B02		480	477	481	4
B03		500	497	501	4
B04	Green	520	517	522	5
B05		536	534	538	4
B06		550	548	552	4
B07		566	564	567	3
B08		580	577	582	5
B09		596	592	598	6
B10		610	607	611	4
B11	Red	626	623	627	4
B12		640	637	641	4
B13		656	653	657	4
B14		670	668	671	3
B15		686	683	687	4
B16	Red Edge	700	697	701	4
B17		716	712	718	6
B18		730	727	731	4
B19		746	743	748	5
B20	Near-infrared	760	756	762	6
B21		776	773	778	5
B22		790	787	791	4
B23		806	802	807	5
B24		820	817	822	5
B25		836	832	839	7
B26		850	846	852	6
B27		866	863	867	4
B28		880	878	884	6
B29		896	894	899	5
B30		908	905	910	5
B31		926	923	927	4
B32		936	933	938	5

**Table 2:** Ranges and distributions of PROSAIL input parameters for the simulated datasets generation.

Parameter	Variables	Unit	Max	Min	Average	Std.	Type
PROSPECT (Leaf parameters)	N	—	2	1	1.5	1	Gaussian
	Cab	μg.cm^−2^	90	5	50	40	Gaussian
	Car	μg.cm^−2^	20	1	10	7	Gaussian
	Cw	cm	0.05	0.001	0.02	0.025	Gaussian
	Cm	g.cm^−2^	0.02	0.001	0.01	0.01	Gaussian
SAIL (Canopy + environment parameters)	LAI	m^2^/m^2^	8	0.001	3.5	2.5	Gaussian
	ALIA	degree	80	30	60	20	Gaussian
	hspot	—	1	0	0.45	0.6	Gaussian
	psoil	—	1	0	0.5	0.5	Gaussian

**Table 3:** Relationship between band combinations and number of bands.

Band Combinations	Number of Bands	Band Combinations	Number of Bands	Band Combinations	Number of Bands
B1B2	C2	B1B2B3B4B14B5B15B13B12B11B10B9	C12	B1B2B3B4B14B5B15B13B12B11B10B9B6B8B16B29B30B27B28B7B31B32	C22
B1B2B3	C3	B1B2B3B4B14B5B15B13B12B11B10B9B6	C13	B1B2B3B4B14B5B15B13B12B11B10B9B6B8B16B29B30B27B28B7B31B32B26	C23
B1B2B3B4	C4	B1B2B3B4B14B5B15B13B12B11B10B9B6B8	C14	B1B2B3B4B14B5B15B13B12B11B10B9B6B8B16B29B30B27B28B7B31B32B26B25	C24
B1B2B3B4B14	C5	B1B2B3B4B14B5B15B13B12B11B10B9B6B8B16	C15	B1B2B3B4B14B5B15B13B12B11B10B9B6B8B16B29B30B27B28B7B31B32B26B25B24	C25
B1B2B3B4B14B5	C6	B1B2B3B4B14B5B15B13B12B11B10B9B6B8B16B29	C16	B1B2B3B4B14B5B15B13B12B11B10B9B6B8B16B29B30B27B28B7B31B32B26B25B24B23	C26
B1B2B3B4B14B5B15	C7	B1B2B3B4B14B5B15B13B12B11B10B9B6B8B16B29B30	C17	B1B2B3B4B14B5B15B13B12B11B10B9B6B8B16B29B30B27B28B7B31B32B26B25B24B23B22	C27
B1B2B3B4B14B5B15B13	C8	B1B2B3B4B14B5B15B13B12B11B10B9B6B8B16B29B30B27	C18	B1B2B3B4B14B5B15B13B12B11B10B9B6B8B16B29B30B27B28B7B31B32B26B25B24B23B22B21	C28
B1B2B3B4B14B5B15B13B12	C9	B1B2B3B4B14B5B15B13B12B11B10B9B6B8B16B29B30B27B28	C19	B1B2B3B4B14B5B15B13B12B11B10B9B6B8B16B29B30B27B28B7B31B32B26B25B24B23B22B21B17	C29
B1B2B3B4B14B5B15B13B12B11	C10	B1B2B3B4B14B5B15B13B12B11B10B9B6B8B16B29B30B27B28B7	C20	B1B2B3B4B14B5B15B13B12B11B10B9B6B8B16B29B30B27B28B7B31B32B26B25B24B23B22B21B17B20	C30
B1B2B3B4B14B5B15B13B12B11B10	C11	B1B2B3B4B14B5B15B13B12B11B10B9B6B8B16B29B30B27B28B7B31	C21	B1B2B3B4B14B5B15B13B12B11B10B9B6B8B16B29B30B27B28B7B31B32B26B25B24B23B22B21B17B20B19	C31

**Table 4:** LAI inversion comparison between the optimal band combination and the full band combination.

	Full-Band	B1B2B4B14B5B15B13B29B19
R^2^	0.63	0.60
RMSE	1.17	1.22
CV	32%	33%

**Table 5:** Correlation between the optimal band combination and LAI.

R	B1	B2	B4	B5	B13	B14	B15	B19	B29	LAI
**B1**	1									
**B2**	0.9998 **	1								
**B4**	0.9101 **	0.9156 **	1							
**B5**	0.8229 **	0.8298 **	0.9593 **	1						
**B13**	0.8165 **	0.8212 **	0.8075 **	0.8437 **	1					
**B14**	0.8572 **	0.8607 **	0.8166 **	0.8244 **	0.9924 **	1				
**B15**	0.8266 **	0.8310 **	0.8092 **	0.8377 **	0.9990 **	0.9959 **	1			
**B19**	0.0960 **	0.0981 **	0.2951 **	0.4416 **	0.1425 **	0.1032 **	0.1338 **	1		
**B29**	−0.0458 **	−0.0452 **	0.0980 **	0.2074 **	−0.0362 **	−0.0644 **	−0.0386 **	0.8868 **	1	
**LAI**	−0.6207 **	−0.6190 **	-0.5167 **	-0.4330 **	−0.4987 **	−0.5375 **	−0.5087 **	0.1932 **	0.3713 **	1

**: highly significant at *p* = 0.05.

## Data Availability

Not applicable.
